# Strengthening Government Leadership in Family Planning Programming in Senegal: From Proof of Concept to Proof of Implementation in 2 Districts

**DOI:** 10.9745/GHSP-D-16-00250

**Published:** 2016-12-23

**Authors:** Barry Aichatou, Cheikh Seck, Thierno Souleymane Baal Anne, Gabrielle Clémentine Deguenovo, Alexis Ntabona, Ruth Simmons

**Affiliations:** a Chief Medical Officer, Diamniadio District, Diamniadio, Senegal. Now Chief Medical Officer, Matam Medical Region, Matam, Senegal.; b Initiative Sénégalaise de Santé Urbaine (ISSU), Dakar, Senegal. Now a consultant, Dakar/Saint-Louis, Senegal.; c ISSU, Dakar, Senegal. Now with The Challenge Initiative, IntraHealth International, Dakar, Senegal.; d ExpandNet, Kinshasa, Democratic Republic of the Congo.; e ExpandNet, Davis, California, USA.

## Abstract

Given Senegal's limited resources, the country receives substantial support from externally funded partner organizations to provide family planning and maternal and child health services. These organizations often take a strong and sometimes independent role in implementing interventions with their own structures and personnel, thereby bypassing the government district health system. This article presents findings from the *Initiative Sénégalaise de Santé Urbaine* (ISSU) (Senegal Urban Health Initiative) that assessed in 2 districts, Diamniadio and Rufisque, the extent to which it was feasible to create stronger government ownership and leadership in implementing a simplified package of family planning interventions from among those previously tested in other districts. The simplified package consisted of both supply- and demand-side interventions, introduced in October 2014 and concluding at the end of 2015. The interventions included ensuring adequate human resources and contraceptive supplies, contraceptive technology updates for providers, special free family planning service days to bring services closer to where people live, family planning integration into other routine services, household visits for family planning education, religious sermons to clarify Islam's position on family planning, and radio broadcasts. District leadership in Diamniadio and Rufisque were actively involved in guiding and implementing interventions, and they also contributed some of their own resources to the project. However, reliance on external funding continued because district budgets were extremely limited. Monitoring data on the number of contraceptive methods provided by district facilities supported by a sister project, the Informed Push Model project, indicate overall improvement in contraceptive provision during the intervention period. In Diamniadio, contraceptive provision increased by 43% between the 6-month period prior to the ISSU interventions (November 2013 through April 2014) and a 6-month intervention period (November 2014 through April 2015), from about 8,000 units to nearly 12,000 units. In Rufisque, contraceptive provision increased by 30%, from more than 17,000 units to more than 22,000 units. Couple-years of protection provided in Diamniadio increased by 82% and in Rufisque by 56%. The experience in these 2 districts in Senegal suggests that it is feasible for districts to play a leadership role in implementing family planning services and mobilizing some of their own resources and that international projects can facilitate capacity building and sustainability within public-sector systems.

## Abstract (precis)

Based on a previous pilot experience, in a next proof-of-implementation phase, district authorities enthusiastically assumed leadership and mobilized local resources to implement a simplified package of family planning interventions, with outside technical support. Comparing a 6-month baseline period with a 6-month implementation period, couple-years of protection increased from about 2,000 to about 4,000 (82% increase) in one district, and from nearly 6,000 to about 9,000 (56% increase) in the second. Longer implementation periods could further support institutionalization and sustainability.

## INTRODUCTION

The literature on implementation science and scale up argues that unless global health projects implement interventions within routine public- or private-sector systems, and with at least a significant portion of their own resources, accomplishments will be short-lived and will at best benefit only a limited number of people.^1^ As Madon and colleagues argue, "Many evidence-based innovations fail to produce results … largely because their implementation is untested, unsuitable or incomplete."^2^

The *Initiative Sénégalaise de Santé Urbaine* (ISSU) (Senegal Urban Health Initiative), an urban reproductive health project implemented initially in 10 urban districts of Senegal, addressed these scale-up issues when authorities from the Dakar Medical Region approached the project to add the 2 remaining districts of the region, Diamniadio and Rufisque, to the project. ISSU used this opportunity to conduct a proof of implementation, assessing whether it was feasible for district authorities to lead the introduction of a simplified package of interventions, partially with their own resources. Interventions were selected from among those previously tested by ISSU with strong project leadership and a substantial amount of external resources in the 10 other districts. In initiating this new approach in the 2 new districts, the ISSU project took a major step toward preparing the ground for future scale up.

ISSU conducted a proof of implementation to assess whether it was feasible for district authorities to lead the introduction of a simplified package of interventions with some of their own resources.

ISSU was a 6-year project (2010–2015), funded by the Bill & Melinda Gates Foundation, which sought to significantly increase Senegal's low contraceptive prevalence rate of 12% in 10 urban districts—8 in the Dakar Medical Region and 2 in other regions. ISSU supported the Ministry of Health and Social Action in its effort to strengthen the country's family planning program, with the ultimate goal of reducing high rates of maternal and infant mortality.^3^ The project's focus on the urban population sought to address the high rates of unmet need for family planning in a country with one of the highest rates of urbanization in the region and one of the lowest contraceptive prevalence rates.^4^^–^^8^ The project was led by IntraHealth International working in a consortium with partner organizations to introduce a broad range of interventions to improve service delivery, community-based interventions, and advocacy for family planning. ISSU played a leading role in developing interventions, as well as in financing and guiding their implementation. An evaluation conducted in 6 districts using a longitudinal survey showed increases in modern contraceptive use between baseline and endline, with baseline rates among all women ranging from 13%–19% and endline rates ranging from 19%–32%.^9^ The initiative in Diamniadio and Rufisque, which ISSU began in its last 2 years of the project cycle, represented a departure from such a project-driven approach.

## FROM PROOF OF CONCEPT TO PROOF OF IMPLEMENTATION

Three developments in the ISSU project led to the decision to assess the feasibility of scaling up the project to 2 additional districts. First, the ISSU team had been introduced to ExpandNet's principles and approaches of scaling up, which emphasized that it is insufficient to provide only a proof of concept, i.e., to prove that a well-implemented package of interventions can achieve substantial results.^10^^–^^12^ Rather, ExpandNet, along with others in the scale-up field, argue that "we are faced with the challenge of determining how the conditions needed for effectiveness can be met within the real-world constraints of health systems operating at large."^13^ In other words, we need proof of implementation demonstrating how successfully tested interventions could be implemented under the leadership and with the resources of the organizations that are intended to scale them up in a routine program context. A proof of implementation is the first step toward ensuring future sustainable, large-scale expansion and institutionalization of tested interventions.

Successfully tested interventions need to be implemented under routine program conditions to assess future sustainability and expansion.

Second, the project had previously undertaken an exercise referred to as capitalization. This exercise involved a systematic analysis to determine the most successful interventions undertaken by ISSU in the original 10 districts which had high potential for sustainable implementation within the national family planning program. The analysis used 4 criteria: relevance, effectiveness, efficiency, and sustainability. This exercise resulted in a smaller package of interventions that became the focus of subsequent activities in the 10 original project districts. The process of capitalization had prepared the project team to further reduce the package of interventions, together with district authorities, to facilitate implementation with fewer resources within the routine program in Diamniadio and Rufisque but with greater involvement of district leadership, mobilization of district resources, and better synergy among externally funded partner organizations.

Third, following dissemination of evidence from the midterm evaluation showing that substantial improvements in contraceptive prevalence had been achieved, the national Directorate of Reproductive Health and Child Survival and the Directorate of the Dakar Medical Region asked ISSU to add the 2 remaining districts of the Dakar Medical Region to the project. The intent was to help these 2 districts reach their expected achievements in contraceptive use.

The 2 contiguous districts are situated 25 km east of Dakar capital. Their population has grown substantially in the past 2 years with a combined population of over half a million. They are predominantly urban but also contain a rural portion. Their economic activities consist of mining, fisheries, poultry farming, vegetable cultivation, and tourism. The public-sector health system in the 2 districts has fewer human and financial resources than the other districts of Dakar Medical Region.

Innovative project-led approaches, such as those pioneered by ISSU, can demonstrate the "efficacy" of interventions. Where the need for these interventions persists beyond the life of a project—as is the case with family planning in Senegal—finding ways of institutionalizing them within the government systems is a critical step toward ensuring that people's needs will continue to be met. Institutionalizing interventions locally can also be a first step toward building capacity within a health system to scale up successful family planning interventions to benefit more people and to foster policy and program development on a sustainable basis.

The purpose of this article is to describe the approach that ISSU used when working in Diamniadio and Rufisque with district health authorities to transition the project from a proof of concept to proof of implementation, that is assessing the extent to which district health authorities were capable of leading implementation of the family planning interventions and reaching the desired outcomes related to service delivery improvements and family planning uptake.

## METHODS

This article uses a case study methodology to assess changes in the mode of implementation as ISSU moved from an initial phase of multiple interventions and strong project leadership by an international NGO to a second phase with a simplified intervention package with strong district leadership. The case study is based on participant observation by the authors, representing the ISSU team, ExpandNet, and district leadership. Collectively these participant observers represent senior Senegalese and external experts with many years of experience in support of family planning and reproductive health project development in Senegal and elsewhere. Their insights were complemented by data gathered from the following sources:

- Informal interviews conducted over the course of the project by the ISSU team and ExpandNet in Diamniadio and Rufisque with the chief medical officers of the districts, reproductive health coordinators, health educators, community health worker (CHW) supervisors, providers, and religious leaders
- The diagnostic assessment undertaken in Diamniadio and Rufisque to determine which supply- and demand-side interventions should be included in the simplified and reduced package
- A simple qualitative tool that documented increased leadership by the district health management teams in the areas of planning, monitoring, supervision, coordination among partners, and mobilization of health systems resources, as well as the changing role of ISSU facilitators
- District-level monitoring related to implementation of the simplified package of interventions (based on CHW records of household visits, the number of referrals from communities for family planning services, and outputs from the special service days) as well as other relevant government service statistics
- Information on the provision of contraceptive products at health facilities collected by the Senegal Informed Push Model project, which focused on improving the contraceptive logistics system^14^

## ISSU'S APPROACH TO WORKING IN DIAMNIADIO AND RUFISQUE

Given that the major purpose of the work in Diamniadio and Rufisque was to assess whether the districts could provide greater leadership in implementing family planning innovations, different approaches were needed from those used in the original ISSU-supported districts. This new approach consisted of 4 key elements: (1) conducting a diagnostic assessment; (2) strengthening district ownership; (3) changing ISSU's role from leadership to facilitation and technical assistance; and (4) creating synergies with partner organizations.

### Conducting a Diagnostic Assessment

The purpose of the diagnostic assessment was to identify:

- A reduced and simplified package of interventions with significant potential to contribute to improved family planning performance, from among those identified in the capitalization process
- The District Health Management Team's (DHMT's) interest and ability to lead the initiative
- The existing conditions of service delivery, particularly the training and supervision needs for family planning
- The needs and perspectives of the community
- The availability and interest of other partners who could assist with family planning program implementation

This diagnostic assessment was essential because Diamniadio and Rufisque had not been included in the diagnostic assessment of the original ISSU study, which had included a population-based longitudinal baseline survey. The assessment in Diamniadio and Rufisque, undertaken in the early months of 2014, was conducted by ISSU with participation from both the DHMT and the Dakar Medical Region.

### Strengthening District Ownership

The country receives substantial support from externally funded partner organizations to implement its family planning and maternal and child health programs, and these organizations not only finance and provide technical support to the programs but also take on a strong and at times independent role in implementing interventions with their own structures and personnel. As a consequence, involvement of district authorities often remains limited. As has been discussed in the literature, this leads to little ownership, limited strengthening of the district health system, and lack of sustainability of the results over the longer term.^15^

Thus, a key objective of the initiative in Diamniadio and Rufisque was to empower the districts to lead the initiative and to assess the extent to which local authorities could guide and supervise the implementation of selected interventions to improve access to quality family planning services while also providing at least some of the resources needed.

### Changing ISSU's Role

Creating district ownership implied a change in the role of ISSU. Although ISSU closely coordinated activities with the DHMTs in the original project districts, it played a strong leadership role by using the human resources and structures of consortium partners to manage and at times implement activities. For example, Marie Stopes International, the *Association Nationale des Sages Femmes d'Etat du Sénégal* (ANSFES) (Senegal's national organization of midwives), and Environment and Development Action in the Third World (ENDA) (an international nonprofit organization), provided clinical outreach services in the communities. ISSU's coordinator for each district played the lead role in ensuring that interventions were coordinated, appropriately implemented, and documented.

In contrast, in Diamniadio and Rufisque the approach was to encourage a shift in ISSU's role from leadership to facilitation and technical assistance. It was clear from the outset, however, that a substantial amount of financial and technical resources from the ISSU project would still be required to implement activities. Such external support was particularly needed during the initial phase of implementation, but it was expected that ISSU support would gradually diminish over time. Examples of costs that needed to be covered externally included:

- Contraceptive technology updates for district providers
- Compensation for household visits undertaken by the volunteer CHWs (the so-called *relais*)
- Some costs for the special free family planning service days; these included the medical supplies associated with contraceptive service provision (which patients themselves had to pay for during routine service delivery but which were waived when services were provided free of charge), payments for lunch, and special compensation for midwives and CHWs
- Training for *imams* (prayer leaders) and for religious relais (community workers who support *imams)* conducted by the *Réseau Islam et Population* (the Islam and Population Network) and the small payments to *imams* for sermons and community talks supporting family planning
- Broadcasts on the local radio station

### Creating Synergy With Partner Organizations

Externally funded organizations tend to work in isolation from each other and often in isolation from the district authorities. A key component of the overall effort was therefore to identify which partners working in the 2 districts were willing to change this pattern and support the initiative.

## THE INTERVENTIONS

From among the package of successful ISSU interventions selected through the capitalization process, ISSU and the DHMT chose an even smaller set of interventions to implement and adapt through a participatory process led by the DHMT (Box). Results from the diagnostic assessment, along with consideration of the capacity of the district to manage certain interventions, were major factors in this selection process. Both supply- and demand-side components were selected.

The simplified package of family planning interventions selected for implementation in Diamniadio and Rufisque consisted of both supply- and demand-side components.

BOX. **The Simplified Intervention Package Implemented in Diamniadio and Rufisque Districts of Senegal and Interventions From the Main ISSU Package Not Implemented**

**Simplified Intervention Package**

Note: In addition to reducing the number of interventions from the main ISSU package, the simplified package of interventions was implemented with less intensity than in the original ISSU project districts.

- Existing midwives assume increased responsibility
- Contraceptive supplies ensured through the Informed Push Model project
- Family planning technical updates for providers
- Free special family planning service days organized by district authorities take the place of mobile outreach from partner organization
- Integration of family planning with other services
- Family planning educational home visits by CHWs
- Family planning talks by Muslim scholars
- Family planning radio broadcasts

**ISSU Interventions Not Implemented**

- Recruitment of additional midwives
- Quality assurance committees
- Mobile clinic outreach organized by partner organization
- Private-sector interventions
- Community-based distribution of oral contraceptives
- Conversations with community groups
- Theater forums
- TV spots
- Work with journalists

Abbreviations: CHW, community health worker; ISSU, *Initiative Sénégalaise de Santé Urbaine* (Senegal Urban Health Initiative).

### Supply-Side Interventions

- **Basic supply-side preconditions consisting of human resources and contraceptive supplies:** Two interventions in the original package were considered to be basic prerequisites without which other interventions should not be started: (1) ensuring adequate human resources including the recruitment of additional midwives, and (2) the availability of contraceptive supplies. Recruitment of additional personnel in Diamniadio and Rufisque was neither feasible nor sustainable, so existing district midwives made a commitment to assume the additional burden of work. Regular availability of contraceptive supplies was ensured through the Informed Push Model, a project designed to improve the contraceptive supply chain.^14^ This project had evolved from ISSU, and its interventions were already being expanded more broadly in Senegal, including in Diamniadio and Rufisque. Thus, ensuring contraceptive supplies required no additional effort from the 2 districts.
- **Contraceptive technology updates for all 51 providers:** In accordance with guidance from the Ministry of Health, the DHMT, with support from ISSU and some assistance from the Dakar Medical Region, organized the contraceptive technology updates which included both didactic and practical components.
- **Special family planning service days:** In ISSU's original 10 project districts, major efforts were made to bring family planning services to the urban poor by providing services free of charge and closer to their areas of residence. This was achieved through collaboration among several ISSU partners involving the use of their mobile clinics and midwives. In Diamniadio and Rufisque, the DHMT used their own limited resources to organize service days at a designated health post or lower-level facility on a rotating basis in poor urban areas where midwives from several nearby health posts joined together to provide free family planning services. Four such special service days were provided per month in each district. A district vehicle was used to transport midwives and needed equipment. Family planning services provided free of charge during these special days included counseling and the full range of available methods comprising oral contraceptives, condoms, intrauterine devices (IUDs), injectables, and implants. CHWs conducted household visits to inform the community of the upcoming special family planning service days.
- **Integration of family planning into other routine services at the health post:** A simple screening tool, also introduced in the original project districts, helped to identify the family planning needs of women of reproductive age who came to the health post to use other services. The tool consisted of 4 basic questions/instructions to assess whether women were ready to adopt a contraceptive method: (1) Do you know about family planning? (2) Are you using contraception? (If yes, advise and thank her.) (3) If not, inform/sensitize her about family planning; and (4) Do you wish to use a contraceptive method? All nurses and midwives of the health posts (including a few from private-sector facilities) in Diamniadio and Rufisque were trained to use this tool by the DHMT, which had received training on this approach from ISSU.

The supply-side interventions that were previously implemented in the original 10 ISSU districts but excluded from Diamniadio and Rufisque consisted of recruitment of additional midwives, quality assurance committees, community-based provision of oral contraceptives, interventions with the private sector, and mobile outreach.

### Demand-Side Interventions

- **Household-based family planning education by CHWs:** Household visits by CHWs to create demand for family planning was introduced by ISSU in Senegal and was identified as a critical need in the diagnostic assessment in Diamniadio and Rufisque. In ISSU's original 10 project districts, groups of CHWs subcontracted by consortium partner organizations made household visits to promote demand for family planning. Adaptations of this approach had to be made to accommodate the implementation realities of the government system in Diamniadio and Rufisque. For example, these visits were assigned to 2 of the CHWs attached to each of the health posts to avoid the need for subcontracting. CHWs are volunteers who exist in all districts in Senegal and are routinely used by various programs to support health interventions with a community mobilization component. They generally receive special training on the subject and a small compensation. The household visits consisted of preparatory contacts with households to schedule a time convenient for the woman to receive the CHW, followed by the visit itself, during which the worker provided family planning education within the broader context of maternal and child health. When appropriate, the CHW referred women to the health post or to a special family planning service day. If needed, the worker made subsequent follow-up visits. CHWs received special training for this activity from the district health team, with technical support from ISSU, and were paid US$1 compensation for each visit to cover their transportation costs. Diamniadio and Rufisque had fewer CHWs than the original 10 districts. Because these were community volunteers rather than full-time paid workers, they were expected to make only 20–30 visits per month on average. Another key adaptation was that the visits were planned together with the chief nurse of the health post. Arrangements were made to ensure each worker was supervised on a monthly basis for monitoring the quality of interactions with women, data collection, and reporting.
- **Islamic sermons and talks on child spacing:** The purpose of this intervention was to clarify the position of Islam on child spacing in the course of sermons, which precede Friday prayer in the large mosques. The intervention was organized in collaboration with key DHMT members and with the Islam and Population Network, the only other ISSU consortium partner participating in Diamniadio and Rufisque. The Network provided training to the *imams* and religious relais as well as materials on family planning and Islam developed by ISSU.
- **Radio broadcasts:** The broadcasts included brief radio spots or musical entertainment on family planning.

ISSU's demand-side interventions that were not included in Diamniadio and Rufisque consisted of conversations with community groups, theater forums, activities with journalists, and TV spots. Moreover, the included interventions were more limited in numbers than what was implemented in the original project districts.

## FINDINGS

### Strengthening District Ownership and Changing Roles of ISSU and Partners

Activities in Diamniadio and Rufisque began between February 2014 and April 2014 with the development of the baseline assessment protocol, and they ended at the same time as the ISSU project at the end of 2015. During the course of this work, the roles of ISSU and the DHMT changed substantially, demonstrating the possibility for a project to make progress toward transferring ownership to district authorities and transitioning to sustainability and eventual broader scale up.

**Empowerment of district authorities:** From the outset, the chief medical officer and other members of the DHMT who participated in the discussions with ISSU were pleased to take an active role in guiding the project. One of the members of the DHMT expressed her enthusiasm about working in this new way by stating:

ISSU came and asked us to take on ownership of the interventions and not only to implement them. We have never had the opportunity to be involved in the conception of a project. We have always been working like robots.

There was never any expectation of special remuneration for this increased level of leadership responsibility because the project helped them reach their own program objectives of increasing contraceptive prevalence. Although district authorities were eager to assume ownership of the initiative, some members of the team were initially worried whether, given the additional burden of work, they would be able to take on this role. However, with time their confidence and creativity to handle the new activities grew, and they were convinced that they could take charge and lead the interventions.

Such confidence and creativity are reflected in how the district team organized itself to take charge and manage the new activities. Key in this process was the team's decision to engage in a broad-based participatory approach to plan and coordinate the family planning innovations that coincided with the development of their annual work plan. In this process they began the systematic use of data for decision making. They committed themselves to monitoring the changes occurring during implementation of new interventions, to analyze results, and to use them to further refine the interventions. As one chief medical officer said:

The DHMT has organized itself to benefit from what ISSU has provided us. We have divided the labor and assigned responsibilities to each person.

One of the key objectives was to mobilize, to the extent possible, district resources. This was primarily feasible in regard to the human resources, and in particular in terms of the midwives of the health posts who joined forces to provide services for the special family planning days. Similarly, the districts also provided vehicles and gasoline when needed to transport midwives and equipment to the places where the free service days were organized. These additions were not major, but they demonstrated that with the motivation generated by encouraging local authorities to take the lead in integrating innovations into the health system, some additional resources could be generated from within.

District authorities mobilized local resources for the project, including human resources and transportation means.

More generally, the DHMT took full responsibility for organizing the special family planning service days. They scheduled these days for the whole district; coordinated the human resources and other logistics, and integrated them into their monthly work plans.

The district team also took charge of organizing the CHWs to implement the family planning educational household visits and to report to the district. Recognizing that the head nurse of the health posts would not be able to adequately supervise the CHWs, the team identified supervisors from among the CHWs who could take charge of this task. This new form of supervision made it feasible to integrate family planning data collected by the CHWs into district service statistics and to include them in district reports.

Another impressive development was that the district teams began to strategize about how the gains made with support from ISSU in Diamniadio and Rufisque could be maintained in the future. Regular meetings between the DHMT, key providers and CHWs, the ISSU team, and other stakeholders were held. In this participatory process, the district began to reorganize available resources and mobilize new ones. For example, the team considered how the local branch of ANSFES could be involved and how resources could become available through the contributions of local health committees.

A chief medical officer best expressed the overall change experienced by members of the district teams:

The approach and the methodology are good because they allow us to be better organized in terms of work and activities. Before, there was insufficient supervision, and not enough attention to what the providers and above all the community workers did. But once the package was there, we could accomplish many things, due to better organization and coordination. Thus, we now have good involvement of key actors in the fight against maternal and infant mortality and for birth spacing.

Nonetheless, there were also limits to the extent to which the districts took ownership of the simplified package of interventions. In particular, there were frequent scheduling conflicts at the district level due to the multiplicity of health programs that had to be coordinated and inadequate human resources in terms of the DHMT, chief nurses at the health post, and midwives.

The team noted that a stronger level of support in the process from the higher-level health management team of the Dakar Medical Region would have been appreciated, although the regional reproductive health coordinator participated in several key initial activities, including one of the training sessions.

**Changing role of ISSU district coordinators:** Empowering the district team to take ownership of the interventions in Diamniadio and Rufisque required a change in the role of the ISSU district coordinators. The 2 coordinators had previous responsibility in other ISSU districts where they provided strong leadership in the implementation of interventions.

For the district team to take on ownership, the coordinators were now required to step back and learn how to act as facilitators or coaches. They had to ensure that both the knowledge and the skills they had acquired previously in other ISSU-supported districts were transferred to the district health team. At the same time, the coordinators had to provide the needed encouragement or even pressure to the district team to move the interventions forward so that the leadership role would not fall back on their shoulders. For example, one of the district health team members mentioned that the ISSU coordinator pushed her to find supervisors for the CHWs. In many other cases, the coordinators deliberately stepped back and referred any inquiries or supervisory issues to the district health team, thereby ensuring that leadership rested with the district.

Project coordinators from ISSU referred any inquiries or supervisory issues to the district health team to facilitate district leadership.

Overall, the coordinators succeeded in transferring their knowledge and skills effectively to the district teams. This was a reflection of the deep commitment of the district teams to demonstrate that they could take on the leadership of this initiative.

**Synergy with other partner organizations:** As previously discussed, several partner organizations worked independently of the DHMT and each other in the districts on various reproductive health and health interventions. As part of their effort to assume greater leadership, the DHMT used their district coordination meetings to encourage collaboration. This effort was successful with regard to other projects from IntraHealth International and from ChildFund International. Thus, a close partnership existed with the Informed Push Model project during the diagnostic assessment and in terms of follow-up for contraceptive supplies as well as with the *Projet de Renforcement des Prestations de Services (*Health Services Integration Project), also led by IntraHealth International. The latter conducted the contraceptive technology updates for providers and supplied the necessary equipment for service delivery points, including insertion and removal equipment for long-acting methods. However, other partner organizations working in the districts did not join the collaboration.

### Demand-Side Interventions

The quantitative results presented here cover the period from November 1, 2014, through April 30, 2015.

**Household visits:** The district team, together with the ISSU coordinators, had targeted a total of 6,000 household visits to be conducted by the 50 CHWs in each of the districts during the 6-month implementation period (20 visits per month per CHW). Actual performance was considerably higher, reaching more than 7,000 visits in each district (Table 1). The CHWs averaged 27 visits per month in Diamniadio and 24 in Rufisque. In addition to contacts with women, the CHWs also reached men during these household visits with brief conversations about family planning.

**TABLE 1.** Family Planning Household Visits and Service Referrals by District, Senegal (November 1, 2014 – April 30, 2015)

Activities	Diamniadio			Rufisque		
	Achievement	Target	Performance	Achievement	Target	Performance
No. of household visits	7,963	6,000	133%	7,314	6,000	122%
No. of women exposed to family planning messages	11,426	6,000	190%	7,483	6,000	125%
No. of women referred to family planning services	1,672	600	279%	1,439	600	240%

**Figure:**
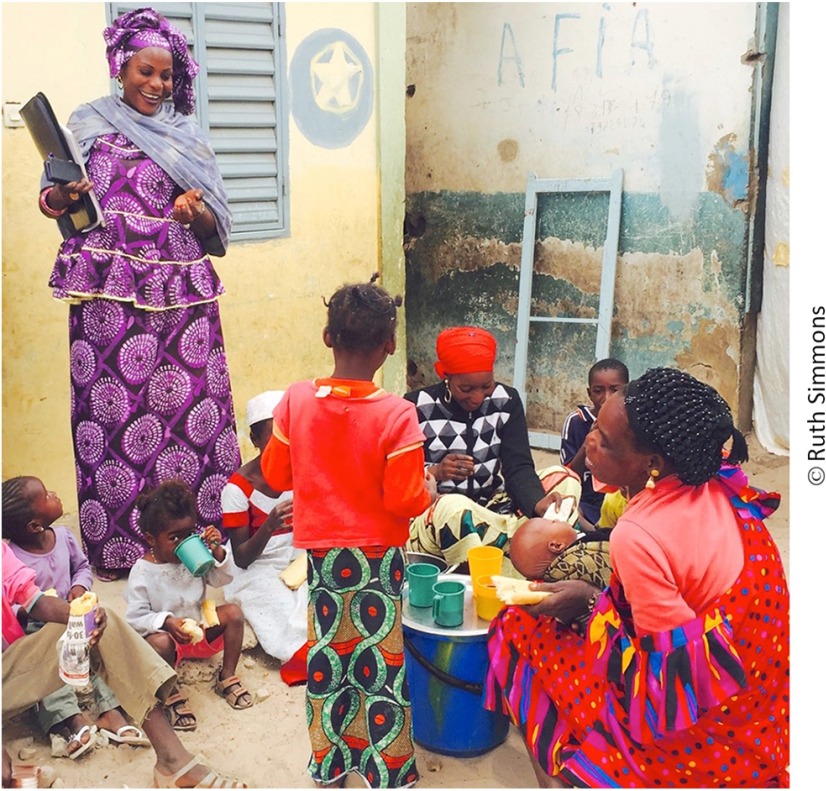
A community health worker (called a *relais* in Senegal) makes household visits to educate families about family planning.

Similarly, service referral for family planning exceeded the targeted number considerably, amounting to over 200% of targets in each district. A major reason why the CHWs exceeded the referral targets was because there was considerably more interest in adopting family planning in these communities than had been anticipated. The CHWs, who received $1 for each household educational family planning visit, were able to satisfy this interest and they provided referrals when needed. Household educational visits did not include community-based contraceptive distribution.

**Sermons and religious conversations:** The 50 *imams* and religious community workers who had been trained by the Islam and Population Network in the 2 districts conducted 14 sermons and 15 religious conversations in Rufisque and 2 sermons and 6 religious conversations in Diamniadio. These relatively low numbers have to be understood in a context where it is still unusual for *imams* to speak publicly about family planning and where support must be built up slowly.

**Radio broadcasts:** The radio station Jokko had been identified by the district teams as having a relevant audience among the local populations of the 2 districts. Therefore, IntraHealth International had signed a contract with the station to transmit family planning messages and together with the district teams they decided the program. In total, Jokko transmitted 5,400 radio spots, 24 musical entertainment segments, and 10 health programs. In the latter, midwives, traditional communicators, and religious community workers as well as religious leaders participated. The following themes were covered in these broadcasts:

- Importance of family planning for infant, child, and maternal health
- Family planning from the perspective of tradition and religion
- Role of political and community leaders and providers in the promotion of family planning
- Treatment of infertility

### Supply-Side Interventions

**Special family planning service days:** Diamniadio held all 4 of the planned special family planning service days per month (24 total over the 6-month implementation period). Rufisque also held most of the planned service days (16 of the planned 24), but not as consistently as Diamniadio. Diamniadio was more successful than Rufisque in recruiting family planning acceptors (total, 768 vs. 470, respectively) (Table 2). Each special family planning day served about 30 new contraceptive users, including a substantial number who selected long-acting methods.

**TABLE 2.** Number of Women Accepting Family Planning During Special Family Planning Service Days, by Method and District, Senegal (November 1, 2014 – April 30, 2015)

	Diamniadio District	Rufisque District
Pills	152	65
Injectables	341	157
Implants	226	195
Intrauterine devices (IUDs)	49	53
**Total number of women**	**768**	**470**
**Couple-years of protection**	**1,180**	**1,028**

Each special family planning day event served about 30 new contraceptive acceptors.

**Integration of family planning into other services:** In total, 139 providers, as well as 9 DHMT members, were trained in the use of the integration screening tool. Data collected between January 2015 and March 2015 indicate that in the 14 facilities of Diamniadio, 375 women were screened with the tool, of whom 104 adopted family planning. In the 24 facilities of Rufisque, 179 of the 2,071 women screened adopted a method. The difference observed between the 2 districts requires further exploration. More generally, it was clear that, similar to the experience within the original ISSU project districts, the providers had considerable difficulties with reporting results from this particular component of the simplified package. In order to address these reporting issues, efforts to incorporate use of the integration questions into registers for prenatal care, postnatal care, curative services, and child care were subsequently piloted.

**Provision of contraceptive methods:** Representative household surveys were not conducted in Diamniadio and Rufisque as was the case in the original ISSU project. Therefore, baseline and endline contraceptive prevalence data are not available for the 2 new districts. However, data on the number and types of contraceptive methods provided by the district facilities covered by the Informed Push Model project are available, from which the couple-years of protection (CYP) can be calculated to provide an estimate of contraceptive coverage. These data confirm the overall improvement in the provision and uptake of family planning services when comparing the 6-month period prior to full implementation of interventions in Diamniadio and Rufisque and a 6-month intervention period 1 year later (Table 3).

**TABLE 3.** Contraceptive Products Provided 6 Months Prior to (November 1, 2013 – April 30, 2014) and 6 Months During Full Implementation of Interventions (November 1, 2014 – April 30, 2015), by District, Senegal

	Diamniadio District			Rufisque District		
	Before	During	% Increase	Before	During	% Increase
Number of contraceptives provided	8,351	11,935	43%	17,551	22,779	30%
Pills	4,540	6,097	34%	9,465	11,867	25%
Injectables	3,568	5,277	48%	7,224	9,373	30%
Implants	187	470	151%	536	1,097	105%
Intrauterine devices (IUDs)	56	91	63%	326	442	36%
Long-acting methods	243	561	131%	862	1,539	79%
Couple-years of protection	2,164	3,932	82%	5,977	9,340	56%

Over this period, in Diamniadio, the number of contraceptives provided increased by 43%, from about 8,000 to nearly 12,000 (Table 3). Rufisque saw a 30% increase in overall contraceptives provided, from more than 17,000 units to more than 22,000 units. Provision of long-acting methods, in particular, increased by 131% in Diamniadio and by 79% in Rufisque. In total, the CYP provided in Diamniadio increased by 82% and in Rufisque by 56% (Table 3). These results reflect accomplishments both with regard to the special free family planning service days, as well as for the increased family planning activity during regular service provision at health posts and other facilities. These results could not have been achieved without the regular availability of contraceptive supplies ensured by the Informed Push Model project.

Couple-years of protection increased by 82% in Diamniadio and 56% in Rufisque as a result of the interventions.

### Cost Containment

Compared with project costs in the original ISSU project districts, considerable cost reductions were achieved in Diamniadio and Rufisque. These reductions were possible for 3 main reasons:

- Reduction in the number of interventions undertaken and the frequency with which they were implemented
- Mobilization of resources from the districts, particularly in the area of human resources
- Increased synergy among IntraHealth International partners supporting the districts in the promotion of family planning activities

Reductions were also achieved in terms of management costs, as well as the institutional and administrative support provided by partners and other community-based organizations in the original project districts. For example, the cost of the special free family planning service days in Diamniadio and Rufisque was only half that of the mobile outreach activity of MSI in the original 10 project districts.

## DISCUSSION

This article is not the first to make a case for strong government ownership and leadership in implementing health innovations.^15^ However, case studies of how to improve family planning services in urban areas and in particular of how to move from a proof of concept to a proof of implementation as demonstrated in Diamniadio and Rufisque are not available for Senegal. Additional initiatives are needed because this case study presents only a beginning. The experience of Diamniadio and Rufisque and its results are encouraging, but questions remain and much needs to be learned including the following:

**Further simplifying the intervention package:** A key question relates to the intervention package—whether it should be further simplified if continued scale up proceeds and whether and how interventions can continue to be improved. As described previously, the package implemented in the 2 new districts resulted from a lengthy process of simplifying a broad range of interventions initially implemented by the ISSU project. The literature on scaling up has pointed out repeatedly that simplifying originally tested interventions is critical for achieving implementation on a larger scale.^11^^,^^12^^,^^16^^,^^17^ Lessons from Diamniadio and Rufisque should be used to assess areas in which further simplification or improvements should be undertaken before scaling up to other areas.

**Institutionalizing interventions:** An important component of scale up is ensuring that interventions are institutionalized in government policies and programs. The project period in Diamniadio and Rufisque was insufficient to focus on such institutionalization. Future efforts to provide a proof of implementation should be conducted over a longer period of time to test the ability of district leadership to assume responsibility for the interventions more systematically and widely. In addition, future efforts should focus on making needed changes in government policies, regulations, and especially budgets to ensure these innovations are sustainable in the long run.

Longer implementation periods can facilitate institutionalization of interventions.

**The role of partners and facilitators:** ISSU coordinators learned a great deal as they shifted toward the role of facilitator, taking a backstage rather than the leading role to which they were accustomed. Efforts to scale up family planning interventions from this experience in Diamniadio and Rufisque or other family planning projects will have to ensure that partner organizations are committed to working in a facilitative rather than dominant role. Moreover, better synergy among partner organizations that support government initiatives is essential, as is commitment from partners and donors to move from proofs of concepts to proofs of implementation. Their commitment to support genuine government ownership is a precondition for future sustainable scale up.

**The need for continued external financial and technical support:** The districts were able to mobilize some of their own resources to implement new family planning interventions, to organize these activities effectively, and to use data for decision making. Nonetheless, major input of external resources, including transportation costs for community health workers, was essential to implement the interventions and to technically support the process. In countries where governments are able to commit more resources to family planning, as is the case in India for example, mechanisms exist for districts to obtain additional funding for the public-sector program. This is not the case in Senegal, and therefore sustainability and scalability of program innovations require external support.

Although the districts mobilized some of their own resources for the project, external resources were still essential.

**The need for longer implementation periods combined with more extensive evaluation:** The current case study presents an important first step in moving toward a pattern where family planning innovations are introduced under the direction and with the resources of the DHMTs. The many questions that remain can only be answered with longer periods of implementation, as well as with more extensive evaluation using both qualitative and quantitative methods.

## CONCLUSION

Among the several outcomes of this case study, none is more important than the extraordinarily positive response from the DHMTs in Diamniadio and Rufisque. Not only were they willing to assume leadership to put in place a range of new family planning interventions, but they were also enthusiastic about the initiative and fully appreciative of the importance of looking for ways to ensure at least some degree of sustainability.

The Diamniadio and Rufisque experience demonstrates that a public-sector system suffering from extensive resource constraints nonetheless contains substantial leadership potential and possibility for mobilizing resources. This potential has been underutilized by externally funded partner organizations who often set up parallel structures to organize family planning services. This is not to imply that such parallel structures are irrelevant. However, government leadership, ownership, and participation must be more deliberately engaged in family planning initiatives at the district level than is often attempted. As Goosby et al. argued in the case of HIV prevention and treatment, "the overall leadership role belongs to the country, not to the external partners."^15^ The case of Diamniadio and Rufisque shows that it is feasible for districts to play this leadership role in implementing family planning innovations in Senegal, to adapt them where needed, and to mobilize at least some resources from within the health system (i.e., to conduct a proof of implementation). The experience demonstrates that international projects can do more than take the lead in organizing effective interventions; they can also facilitate capacity building within public-sector systems to achieve sustainable interventions, even though a considerable level of external resources may still be essential. The family planning needs of women, men, and adolescents would be better served if such an approach were more widely practiced.

Public-sector systems suffering from extensive resource constraints nonetheless have substantial leadership potential.

We hope this experience will receive wide discussion and that similar efforts will be undertaken by others. The results and lessons learned are likely to be highly relevant in other countries in Francophone West Africa with low modern contraceptive use and relatively weak family planning programs that are dependent on external support. Wider adoption of the Diamniadio and Rufisque approach could lead to the much-needed institutionalization and subsequent sustainability of successfully tested family planning interventions in countries of the region.

## Notes

**Peer Reviewed**

**First Published Online:** 2016 Dec 2

## References

[1] Peters DH Tran NT Adam T Implementation research in health: a practical guide. Geneva: Alliance for Health Policy and Systems Research, World Health Organization; 2013 Available from: http://who.int/alliance-hpsr/alliancehpsr_irpguide.pdf

[2] Madon T Hofman KJ Kupfer L Glass RI Implementation science. Science. 2007;318(5857):1728–1729. 10.1126/science.1150009. 18079386

[3] Futures Group. Repositioning family planning in Senegal: status of family planning programs in Senegal. Washington (DC): Futures Group; 2013 Available from: http://www.healthpolicyproject.com/ns/docs/Senegal_WestAfricaBriefs_Final.pdf

[4] Population Reference Bureau (PRB). Senegal. Reproductive transitions: unmet need for family planning. Washington (DC): PRB; 2014 Available from: www.prb.org/pdf14/senegal-unmet-need-contraception.pdf

[5] Sidze EM Lardoux S Speizer IS Faye CM Mutua MM Badji F Young women’s access to and use of contraceptives: the role of providers’ restrictions in urban Senegal. Int Perspect Sex Reprod Health. 2014;40(4):176–184. 10.1363/4017614. 25565345 PMC6652199

[6] Ministry of Health and Social Welfare (MOHSW)[Senegal]. National family planning action plan 2012-2015. Dakar (Senegal): MOHSW; 2012 Available from: http://ec2-54-210-230-186.compute-1.amazonaws.com/wp-content/uploads/2014/10/Senegal_National_FP_Action_Plan_English.pdf

[7] Wickstrom J Diagne A Smith A Senegal case study: promising beginnings, uneven progress. A repositioning family planning case study. New York: The ACQUIRE Project, EngenderHealth; 2006 Available from: http://www.acquireproject.org/fileadmin/user_upload/ACQUIRE/Publications/Senegal-case-study-final.pdf

[8] Jacobstein R Bakamjian L Pile JM Wickstrom J Fragile, threatened, and still urgently needed: family planning programs in sub-Saharan Africa. Stud Fam Plann. 2009;40(2):147–154. 10.1111/j.1728-4465.2009.00197.x. 19662806

[9] Measurement, Learning and Evaluation Project (MLE); Initiative Sénégalaise de Santé Urbaine (ISSU); Agence pour la Promotion des Activités de Population – Sénégal (APAPS); Global Research and Advocacy Group (GRAG). Sénégal 2015 étude finale. Chapel Hill (NC): MLE; 2016 Co-published by ISSU, APAPS, and GRAG. Available from: https://www.urbanreproductivehealth.org/resource/mesure-apprentissage-et-evaluation-de-linitiative-s%C3%A9n%C3%A9galaise-de-sant%C3%A9-urbaine-issu-s%C3%A9n%C3%A9gal

[10] Simmons R Fajans P Ghiron L, editors. Scaling up health service delivery: from pilot innovations to policies and programmes. Geneva: World Health Organization; 2007 Available from: http://www.who.int/immunization/hpv/deliver/scalingup_health_service_delivery_who_2007.pdf

[11] ExpandNet, World Health Organization. Nine steps for developing a scaling-up strategy. Geneva: World Health Organization; 2010 Available from: http://www.expandnet.net/PDFs/ExpandNet-WHO%20Nine%20Step%20Guide%20published.pdf

[12] ExpandNet, World Health Organization. Beginning with the end in mind: planning pilot projects and other programmatic research for successful scaling up. Geneva: World Health Organization; 2011 Available from: http://www.who.int/reproductivehealth/publications/strategic_approach/9789241502320/en/

[13] Birthing centers staffed by skilled birth attendants: can they be effective … at scale? Glob Health Sci Pract. 2016;4(1):1–3. 10.9745/GHSP-D-16-00063. 27016537 PMC4807742

[14] Daff BM Seck C Belkhayat H Sutton P Informed push distribution of contraceptives in Senegal reduces stockouts and improves quality of family planning services. Glob Health Sci Pract. 2014;2(2):245–252. 10.9745/GHSP-D-13-00171. 25276582 PMC4168620

[15] Goosby E Von Zinkernagel D Holmes C Haroz D Walsh T Raising the bar: PEPFAR and new paradigms for global health. J Acquir Immune Defic Syndr. 2012;60 Suppl 3:S158–S162. 10.1097/QAI.0b013e31825d057c. 22797738

[16] Atun R de Jongh T Secci F Ohiri K Adeyi O Integration of targeted health interventions into health systems: a conceptual framework for analysis. Health Policy Plan. 2010;25(2):104–111. 19917651 10.1093/heapol/czp055

[17] Gericke CA Kurowski C Ranson MK Mills A Intervention complexity: a conceptual framework to inform priority-setting in health. Bull World Health Organ. 2005;83(4):285–293. 15868020 PMC2626218

